# Higher platelet cytochrome oxidase specific activity in surviving than in non-surviving septic patients

**DOI:** 10.1186/cc13956

**Published:** 2014-06-30

**Authors:** Leonardo Lorente, María M Martín, Esther López-Gallardo, Ruth Iceta, José Blanquer, Jordi Solé-Violán, Lorenzo Labarta, César Díaz, Alejandro Jiménez, Julio Montoya, Eduardo Ruiz-Pesini

**Affiliations:** 1 Intensive Care Unit, Hospital Universitario de Canarias, Ofra, s/n. La Laguna-38320, Santa Cruz de Tenerife, Spain; 2 Intensive Care Unit. Hospital Universitario Nuestra Señora Candelaria, Crta Rosario s/n, Santa Cruz Tenerife 38010, Spain; 3 Departamento de Bioquímica y Biología Molecular y Celular, Centro de Investigaciones Biomédicas En Red de Enfermedades Raras (CIBERER) e Instituto Aragonés de Ciencias de la Salud, Universidad de Zaragoza, Zaragoza 50013, Spain; 4 Intensive Care Unit. Hospital Clínico Universitario de Valencia, Avda. Blasco Ibáñez nº17-19, Valencia 46004, Spain; 5 Intensive Care Unit, Hospital Universitario Dr. Negrín, Barranco de la Ballena s/n., Las Palmas de Gran Canaria 35010, Spain; 6 Intensive Care Unit, Hospital San Jorge de Huesca, Avenida Martínez de Velasco nº36, Huesca 22004, Spain; 7 Intensive Care Unit, Hospital Insular, Plaza Dr. Pasteur s/n., Las Palmas de Gran Canaria 35016, Spain; 8 Research Unit, Hospital Universitario de Canarias, Ofra, s/n. La Laguna - 38320, Santa Cruz de Tenerife, Spain; 9 Fundación ARAID, Zaragoza, Spain

## Abstract

**Introduction:**

In a previous study with 96 septic patients, we found that circulating platelets in 6-months surviving septic patients showed higher activity and quantity of cytochrome c oxidase (COX) normalized by citrate synthase (CS) activity at moment of severe sepsis diagnosis than non-surviving septic patients. The objective of this study was to estimate whether COX specific activity during the first week predicts 1-month sepsis survival in a larger cohort of patients.

**Methods:**

Using a prospective, multicenter, observational study carried out in six Spanish intensive care units with 198 severe septic patients, we determined COX activity per proteins (COXact/Prot) in circulating platelets at day 1, 4 and 8 of the severe sepsis diagnosis. Endpoints were 1-month and 6-months mortality.

**Results:**

Survivor patients (n = 130) showed higher COXact/Prot (P < 0.001) than non-survivors (n = 68) at day 1, 4 and 8 of severe sepsis diagnosis. More than a half of the 6-months survivor patients showed an increase in their COXact/Prot from day 1 to 8. However, most of the 1-month non-survivors exhibited a decrease in their COXact/Prot from day 1 to 8. Multiple logistic regression analyses showed that of platelet COXact/Prot > 0.30 mOD/min/mg at day 1 (P = 0.002), 4 (P = 0.006) and 8 (P = 0.02) was associated independently with 1-month mortality. Area under the curve of COXact/Prot at day 1, 4 and 8 to predict 30-day survival were 0.70 (95% CI = 0.63-0.76; P < 0.001), 0.71 (95% CI = 0.64-0.77; P < 0.001) and 0.71 (95% CI = 0.64-0.78; P < 0.001), respectively.

**Conclusions:**

The new findings of our study, to our knowledge the largest series reporting data about mitochondrial function during follow-up in septic patients, were that septic patients that survive 1-month have a higher platelet cytochrome oxidase activity at moment of sepsis diagnosis and during the first week than non-survivors, and that platelet cytochrome oxidase activity at moment of sepsis diagnosis and during the first week could be used as biomarker to predict the clinical outcome in septic patients.

## Introduction

Severe sepsis is a fatal condition associated with organ failure [1]. This organ dysfunction is related to tissue hypoxia due to cellular inability to use oxygen [2]. Most cellular oxygen is consumed by cytochrome oxidase (COX) in the electron transport chain (ETC) of the mitochondrial oxidative phosphorylation system (OXPHOS). Thus, in a previous study of 96 patients with severe sepsis, we found that 6-month survivors had higher platelet COX activity normalized by citrate synthase (CS) activity at the time of diagnosis than non-survivors [3]. Because the determination of platelet COX activity to assess ETC is a rapid, easy and not very invasive protocol, this parameter could be used as a biomarker of sepsis mortality. Moreover, patients with low COX activity could benefit from drugs that improve ETC [3].

However, despite the inclusion of 96 individuals as the largest series reported to date in which COX activity has been determined, it is still a relatively small cohort of patients. Moreover, although 6-month survival allows the analysis of long-term outcomes, this period is not the most-used end point [4] because confounder factors unrelated to the initial sepsis event would affect the results. In addition, COX activity was only determined in platelets obtained at the time of diagnosis and potential changes over time were not evaluated. To address these problems, we have now determined COX-specific activity in a larger population of septic patients, analyzed 1-month survival and obtained blood samples not only within the first 24 hours after the diagnosis of severe sepsis but also on days 4 and 8.

## Material and methods

### Design and subjects

A prospective, multicenter, observational, study was carried out in six Spanish ICUs. The study was approved by the Institutional Ethical Review Boards of the six hospitals participating in the study: Hospital Universitario de Canarias (La Laguna. Santa Cruz de Tenerife. Spain), Hospital Universitario Nuestra Señora de Candelaria (Santa Cruz de Tenerife. Spain), Hospital Universitario Dr. Negrín (Las Palmas de Gran Canaria. Spain), Hospital Clínico Universitario de Valencia (Valencia. Spain), Hospital San Jorge (Huesca. Spain) and Hospital Insular (Las Palmas de Gran Canaria. Spain). Written informed consent from the patients or from the family members was obtained.

Inclusion criteria were the diagnosis of severe sepsis according to the International Sepsis Definitions Conference criteria [5]. Exclusion criteria were: age <18 years, pregnancy, lactation, platelet transfusion, HIV, white blood cell count <1,000 cells/μl, solid or hematological tumor, or immunosuppressive, steroid or radiation therapy.

### Variables recorded

The following variables were recorded for each patient: sex, age, diabetes mellitus, chronic obstructive pulmonary disease (COPD), ischemic heart disease, chronic renal failure (defined as glomerular filtration rate (GFR) <60 ml/minute per 1.73 m^2^), site of infection, microorganism responsible, bloodstream infection, empiric antimicrobial treatment, septic shock, pressure of arterial oxygen/fraction inspired of oxygen (PaO_2_/FIO_2_), creatinine, bilirubin, leukocytes, lactic acid, platelets, international normalized ratio (INR), activated partial thromboplastin time (aPTT), acute physiology and chronic health evaluation (APACHE)-II score [6] and sepsis-related organ failure assessment (SOFA) score [7].

### Endpoints

We assessed survival at 1 month and 6 months as the endpoints.

### Biochemical determination in platelets

Blood samples were collected from patients on day 1, 4 and 8 of diagnosis of severe sepsis for the determination of COX. Day 1 was considered as the first day that severe sepsis was diagnosed (baseline values). Day 4 was considered as the day after 72 hours had elapsed, and day 8 as the day after 7 days had elapsed since the diagnosis of severe sepsis.

Platelets were obtained according to previously described protocols [8]. We measured the COX activity (COXact) by using Mitoprofile® Human Complex IV Activity and Quantity from Mitosciences (Invitrogen) according to the manufacturer’s instructions. This kit immunocaptures complex IV and activity is determined colorimetrically by following the oxidation of reduced cytochrome c as an absorbance decrease at 550 nm. Protein levels were assayed following previously described protocols [9], and were expressed in mg. A NovoStar MBG Labtech microplate instrument was used for COXact and protein determinations. COX activity corrected by proteins (COXact/Prot) was expressed as milli-optical density per minute per mg of proteins (mOD/min/mg) multiplied by 100.

### Statistical analysis

Categorical variables are reported as frequencies and percentages and continuous variables are reported as medians and interquartile ranges. Comparisons of categorical variables between survivors and non-survivors were carried out with the chi-square test. Normality assumption was tested with the Komogorov-Smirnov test. Comparisons of continuous variables between survivors and non-survivors were carried out using the Wilcoxon-Mann–Whitney test. We used the Kruskal-Wallis test to compare levels of COXact/Prot between patients grouped by survival lower than 1 month, between 1 and 6 months and more than 6 month; post-hoc comparison of pairs was carried out with the Mann-Whitney test.

Multiple logistic regression analysis was used to determine the independent contribution of platelet COXact/Prot >0.30 mOD/min/mg, lactic acid and SOFA score at day 1, 4 and 8 to the prediction of 1- and 6-month mortality. Odds ratios (ORs) and 95% confidence intervals were calculated as measures of the association. Receiver operating characteristic (ROC) analyses were carried out to determine the goodness-of-fit of the COXact/Prot at day 1, 4 and 8 to predict 1- and 6-month mortality. Analysis of survival with the Kaplan-Meier method curve and comparisons by log-rank test were carried out using COXact/Prot lower/higher than 0.30 mOD/min/mg at day 1, 4 and 8 as the independent variable and survival at 1 and 6 months as the dependent variable. A *P*-value less than 0.05 was considered statistically significant. Statistical analyses were performed with SPSS v. 17.0 (SPSS Inc., Chicago, IL, USA) and Med Calc v. 10.1.3.0 (Mariakerke, Belgium).

## Results

In a previous study of 96 sepsis patients, we found that 6-month survivors had a higher platelet COXact/CSact ratio at the time of diagnosis than non-survivors [3]. This result suggested that OXPHOS function could be used as a biormarker of sepsis mortality. As we found a tight correlation between COXact/CSact and COXact/Prot (Spearman rho = 0.77; *P* <0.001) in these patients, to confirm the association between OXPHOS function and sepsis survival and simplify clinical protocols, we have now studied COXact/Prot in a larger number of individuals (198 sepsis patients). We have observed that COXact/Prot at the time of sepsis diagnosis was significantly higher in 6-month survivors than in non-survivors among patients with sepsis (Table 1).

**Table 1:** **Platelet cytochrome c oxidase activity per proteins *(*COXact**/**Prot) in sepsis patients according to survival or non**-**survival at 1 and 6 months**

**Survival**	**COXact/Prot determination day**	**Survivors**	**Non-survivors**	**P-value**
6-months				
	Day 1	0.27 (0.11, 0.76)	0.13 (0.05, 0.37)	<0.001
		(n = 109)	(n = 89)	
	Day 4	0.29 (0.13, 0.92)	0.11 (0.05, 0.42)	<0.001
		(n = 109)	(n = 71)	
	Day 8	0.59 (0.13, 1.00)	0.09 (0.04, 0.37)	<0.001
		(n = 109)	(n = 58)	
1-month				
	Day 1	0.28 (0.11, 0.61)	0.10 (0.05, 0.26)	<0.001
		(n = 130)	(n = 68)	
	Day 4	0.29 (0.11, 0.92)	0.07 (0.05, 0.24)	<0.001
		(n = 130)	(n = 50)	
	Day 8	0.51 (0.10, 1.00)	0.06 (0.04, 0.25)	<0.001
		(n = 130)	(n = 37)	

Medians (25^th^, 75^th^ percentiles) are indicated.

Although 6-month survival allows the analysis of long-term outcomes [4], confounder factors unrelated to the initial sepsis event could affect the results. Therefore, we have repeated these analyses for 1-month survival because this is a more frequently used parameter in sepsis patients. We found that 1-month survivors of sepsis (n = 130) had lower lactic acid (*P* <0.001), INR (*P* = 0.03), aPTT (*P* = 0.01), APACHE-II score (*P* <0.001) and SOFA score (*P* <0.001), and higher platelet count (*P* = 0.01) at the time of diagnosis of severe sepsis than non-survivors (n = 68). There were no differences in the other recorded variables between survivors and non-survivors of sepsis (Table 2) except in COXact/Prot. Severely septic patients who survived 1 month had significantly higher COXact/Prot at the time of sepsis diagnosis than non-survivors (Table 1). Of 130 patients who survived 1 month, 21 died before 6 months and their COXact/Prot values were not significantly different from those of 109 patients who survived for longer than 6 months (Table 3).

**Table 2:** Characteristics of 1-month survivors and non-survivors among patients with sepsis

	**Survivors (n = 130)**	**Non-survivors (n = 68)**	***P*-value**
Gender, male, n (%)	89 (68.5)	41 (60.3)	0.27
Age, years, median ( 25^th^, 75^th^ percentile)	54 (44, 64)	61 (50, 72)	0.11
Diabetes mellitus, n (%)	33 (25.4)	24 (35.3)	0.19
COPD, n (%)	15 (11.5)	9 (13.2)	0.82
Ischemic heart disease, n (%)	13 (10.0)	7 (10.3)	0.99
Chronic renal failure, n (%)	6 (4.6)	7 (10.3)	0.14
Site of infection, n (%)			0.40
Respiratory	72 (55.4)	40 (58.8)	
Abdominal	39 (30.0)	18 (26.5)	
Neurological	2 (1.5)	1 (1.5)	
Urinary	8 (6.2)	2 (2.9)	
Skin	6 (4.6)	1 (1.5)	
Endocarditis	3 (2.3)	5 (7.3)	
Osteomyelitis	0	1 (1.5)	
Microorganism responsible, n (%)			
Unknown	68 (52.3)	35 (51.5)	0.99
Gram-positive	28 (21.5)	19 (27.9)	0.38
Gram-negative	32 (24.6)	14 (20.6)	0.60
Fungii	3 (2.3)	3 (4.4)	0.42
Anaerobe	1 (0.8)	1 (1.5)	0.99
Bloodstream infection, n (%)	20 (15.4)	11 (16.2)	0.99
Empiric antimicrobial treatment, n (%)			0.89
Unknown if adequate due to negative cultures	65 (50.0)	33 (48.5)	
Unknown if adequate due to diagnosis by antigenuria	4 (3.1)	3 (4.4)	
Adequate	55 (42.3)	30 (44.1)	
Inadequate	6 (4.6)	2 (2.9)	
Betalactamic more aminoglycoside, n (%)	26 (20.0)	16 (23.5)	0.59
Aminoglycoside, n (%)	36 (27.7)	18 (26.5)	0.99
Betalactamic more quinolone, n (%)	70 (53.8)	36 (52.9)	0.99
Septic shock, n (%)	109 (83.8)	62 (91.2)	0.19
PaO_2_/FIO_2_ ratio, median (25^th^, 75^th^ percentile)	159 (105, 260)	168 (109, 237)	0.58
Creatinine, mg/dl , median (25^th^, 75^th^ percentile)	1.10 (0.80, 1.90)	1.60 (0.90, 2.65)	0.08
Bilirubin, mg/dl, median (25^th^, 75^th^ percentile)	0.90 (0.40, 1.55)	0.88 (0.47, 2.54)	0.63
Leukocytes, cells/μl, median*10^3^ (25^th^, 75^th^ percentile)	14.0 (8.6, 21.5)	15.1 (6.4, 20.2)	0.84
Lactic acid, mmol/l, median (25^th^, 75^th^ percentile)	1.80 (1.20, 3.50)	3.80 (1.55, 6.25)	<0.001
Platelets, cells/μl, median*10^3^ (25^th^, 75^th^ percentile)	192 (112, 267)	124 (64, 196)	0.01
INR, median (25^th^, 75^th^ percentile)	1.26 (1.10, 1.50)	1.42 (1.15, 1.68)	0.03
aPTT, seconds, median (25^th^, 75^th^ percentile)	32 (28, 37)	38 (29, 45)	0.01
APACHE-II score, median (25^th^, 75^th^ percentile)	18 (14, 23)	23 (17, 28)	<0.001
SOFA score, median (25^th^, 75^th^ percentile)	9 (7, 12)	11 (9, 14)	0.001

COPD, chronic obstructive pulmonary disease; PaO_2_/FIO_2_, pressure of arterial oxygen/fraction inspired oxygen; INR, international normalized ratio; aPTT, activated partial thromboplastin time; APACHE, acute physiology and chronic health evaluation; SOFA, sepsis-related organ failure assessment.

**Table 3:** **Platelet cytochrome c oxidase activity per proteins *(*COXact**/**Prot) of 6-month survivors, 1-month but non-6-month survivors and 1-month non-survivors**

**Day**	**6MS**	**1MS/6MN-S**	**1MN-S**	***P*-value**
Day 1	0.27 (0.11-0.76)*	0.46 (0.13-0.50)^†^	0.10 (0.05-0.26)	<0.001
	(n = 109)	(n = 21)	(n = 68)	
Day 4	0.29 (0.13-0.92)*	0.25 (0.09-0.71)^†^	0.07 (0.05-0.24)	<0.001
	(n = 109)	(n = 21)	(n = 50)	
Day 8	0.59 (0.13-1.00)*	0.15 (0.08-0.68)	0.06 (0.04-0.25)	<0.001
	(n = 109)	(n = 21)	(n = 37)	

*P-values <0.001 for the comparison between patients who were 6-month survivors (6MS) and 1-month non-survivors (1MN-S); ^†^*P*-values <0.05 for the comparison between patients who were 1-month but non-6-month survivors (1MS/6MN-S) and 1MN-S. The remainder of the paired comparisons were not statistically significant.

In addition, we found that 1-month survivor patients had statistically significantly higher COXact/Prot at days 4 and 8 than non-survivor ones (Table 1).

We found statistically significant differences comparing the proportions of patients with increasing COXact/Prot from days 1 to 8 between patients surviving longer than 6-months, 1 to 6 months, and less than 1 month (55.0% (60/109), 38.1% (8/21), 29.7% (11/37), respectively; *P* = 0.02). Comparing pairs of groups, we found an increased proportion of patients with increasing COXact/Prot from days 1 to 8 among patients surviving longer than 6 months compared with those surviving less than 1 month (*P* = 0.01), and we did not find statistically significant differences between the 6-month survival group and the group that survived 1 month but not 6 months (*P* = 0.23), or between patients who survived 1-month but not 6 months and those who did not survive 1 month (*P* = 0.57).

Supporting the relationship between COXact/Prot or its evolution with sepsis mortality, COXact/Prot at day 1 of 31 patients who did not survive a week (0.06 (0.05-0-18)) was significantly lower (*P* = 0.03) than that of 37 patients that survived more than a week but less than 1 month (0.14 (0.07-0.32)).

Multiple logistic regression analyses showed that of platelet COXact/Proteins >0.30 mOD/min/mg at day 1 (*P* = 0.002), day 4 (*P* = 0.006) and day 8 (*P* = 0.02) was associated independently with 1-month mortality, controlling for lactic acid levels and SOFA score (Table 4).

**Table 4:** Multiple logistic regression analyses to predict mortality at 1 month

	**Odds ratio**	**95% confidence interval**	***P*-value**
**First model**			
Platelet COXact/Prot >0.30 mOD/min/mg at day 1	0.34	0.17, 0.67	0.002
Lactic acid at day 1	1.19	1.05, 1.34	0.004
SOFA score at day 1	1.07	0.98, 1.17	0.13
**Second model**			
Platelet COXact/Prot >0.30 mOD/min/mg at day 4	0.30	0.13, 0.71	0.006
Lactic acid at day 4	1.49	1.06, 2.07	0.02
SOFA score at day 4	1.13	1.03, 1.24	0.01
**Third model**			
Platelet COXact/Prot >0.30 mOD/min/mg at day 8	0.34	0.13, 0.87	0.02
Lactic acid at day 8	1.16	0.75, 1.80	0.51
SOFA score at day 8	1.16	1.06, 1.26	0.001

COXact/Prot, cytochrome c oxidase specific activity; SOFA, sepsis-related organ failure assessment.

**Figure 1:**
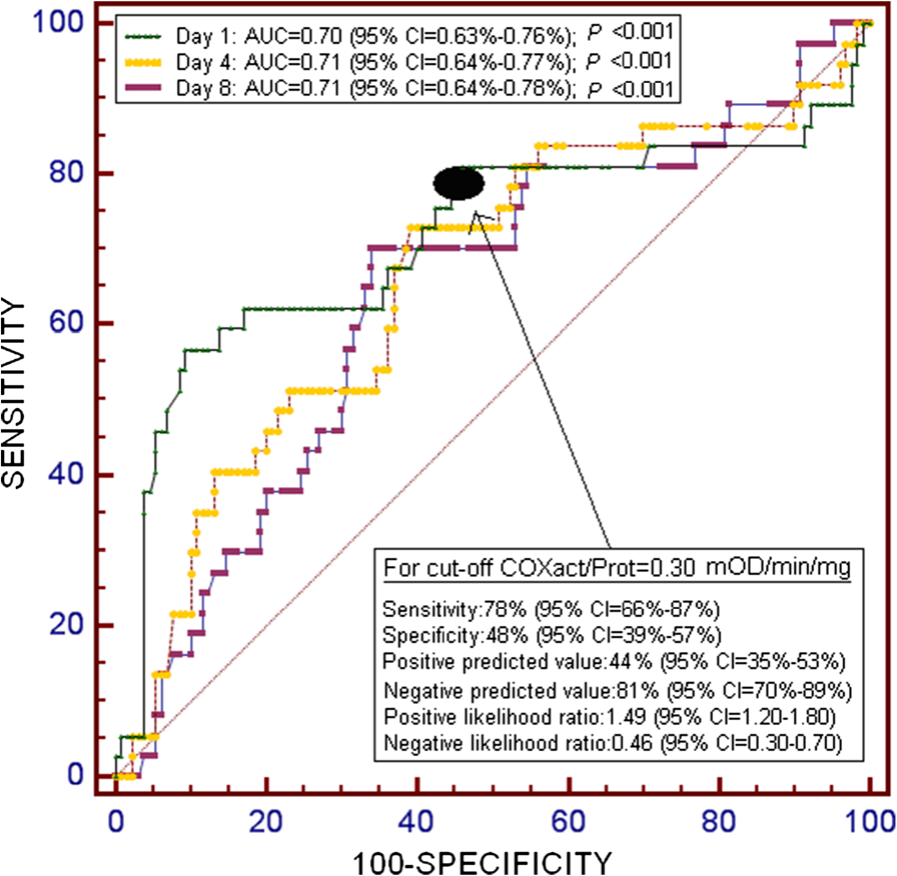
Receiver operation characteristic analyses using platelet cytochrome c oxidase activity per protein (COXact/Prot) at 1, 4 and 8 days after admission as a predictor of mortality at 1 month in septic patients.

**Figure 2:**
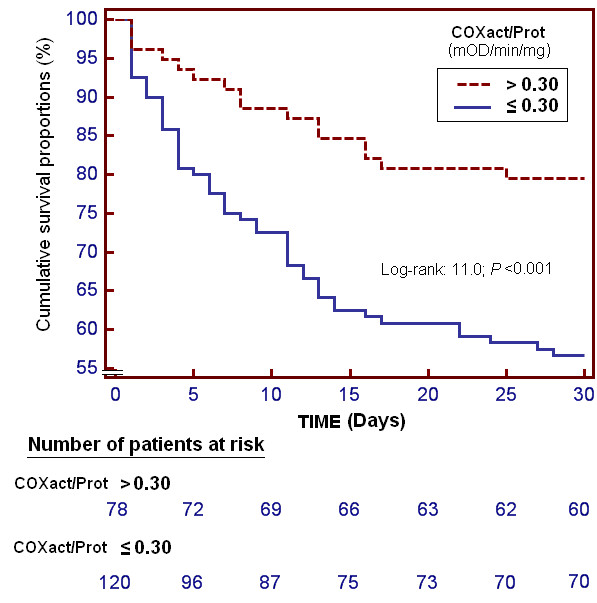
Cumulative proportion of survival septic patients at 1-month using platelet cytochrome c oxidase activity per proteins (COXact/Prot) higher or lower than 0.30 mOD/min/mg of proteins.

Area under the curve of COXact/Prot at day 1, 4 and 8 to predict 1-month survival were 0.70 (95% CI 0.63, 0.76; *P* <0.001), 0.71 (95% CI 0.64, 0.77; *P* <0.001) and 0.71 (95% CI 0.64, 0.78; *P* <0.001), respectively (Figure 1).Kaplan-Meier survival analysis showed that patients with COXact/Prot higher than 0.30 mOD/min/mg of proteins at day 1 (log-rank = 11.0; P < 0.001) (Figure 2), day 4 (log-rank = 11.0; P < 0.001), and day 8 (log-rank = 13.0; P < 0.001) had a higher probability of survival at 1-month than patients with lower COXact/Prot.

## Discussion

Platelets contain small numbers of fully functional mitochondria. Their abundance in blood as well as the relative ease to obtain them with minimally invasive protocols has made platelets an attractive source of human mitochondria. Platelet OXPHOS function has frequently been used to study OXPHOS dysfunction as a biomarker in human disease [10]. COXact/Prot can be used as a surrogate of OXPHOS function. Because COX subunits are lower in platelets than in other blood cells, the detection of a pathology associated with decreased COXact/Prot will be most readily revealed in platelets [11]. Some commercial kits are able to determine COXact/Prot using a microplate reader, an instrument frequently found in the clinical chemistry laboratories from most of the hospitals. Therefore, to verify our previous observation that platelet OXPHOS function can be used as a biomarker of sepsis mortality [3] and simplify clinical protocols, we have now studied the COXact/Prot in a larger number of sepsis patients using a fast and easy test.

We have confirmed that platelet COXact/Prot at the time of diagnosis of sepsis is significantly higher in 1-month survivors than in non-survivors. It would be possible that some individuals have a lower basal OXPHOS capacity and, for them, the sepsis insult may represent an unbearable stress. In fact, we have already observed that there are OXPHOS differences between transmitochondrial cell lines with distinct mitochondrial DNA (mtDNA) genetic backgrounds (haplogroups) obtained from normal individuals [12] and that patients from mtDNA haplogroup JT have a higher platelet OXPHOS function and 1-month survival [13,14].

It has been reported that OXPHOS inhibition can affect the platelet prothrombotic function [15,16]. However, on the contrary, activation of coagulation frequently occurs in sepsis [17]. Therefore, platelet OXPHOS dysfunction observed in sepsis patients probably is not enough to alter platelet function and increase mortality. However, as previously discussed, sepsis provokes mitochondrial dysfunction in different tissues from different species [3]. Thus, the lower platelet COXact/Prot in non-surviving patients probably mirrors a general situation in other tissues and organs and this general lower OXPHOS function would be one of the reasons for death.

It is possible that independently of their basal COXact/Prot, non-surviving patients have an impaired response to the septic insult. COXact/Prot changes along the time. Thus, a higher percentage of 6-month survivors have increased COXact/Prot along the first week compared to patients who do not survive 1 month. An interesting aspect is that the patients who died after 1 month but within 6 months of the sepsis event had an initial COXact/Prot similar to that of those who survived more than 6 months; however, the percentage of patients with increased COXact/Prot along the first week was lower among patients who survived 1 month but died within 6 months of the sepsis compared to those who survived 6 months. If this was the case, early determinations of platelet COXact/Prot along the first week might help to predict the survival probabilities and, for those patients with higher risk of death, the administration of different compounds that increase COXact/Prot may improve their survival chances [18-20].

## Conclusions

Septic patients who survive 1 month have higher platelet cytochrome oxidase-specific activity at the time of diagnosis than non survivors. A decrease in the platelet cytochrome oxidase specific activity along the first week after the sepsis diagnosis is a factor for poor prognosis. Therefore, platelet cytochrome oxidase-specific activity can be used as a biomarker of outcome in septic patients.

## Key messages

• Septic patients who survive 1 month have a higher platelet cytochrome oxidase-specific activity at the time of diagnosis than non survivors.

• A decrease in the platelet cytochrome oxidase-specific activity along the first week after the sepsis diagnosis a factor for poor prognosis.

• Platelet cytochrome oxidase-specific activity can be used as a biomarker of outcome in septic patients.

## Abbreviations

APACHE: acute physiology and chronic health evaluation; aPTT: activated partial thromboplastin time; COPD: chronic obstructive pulmonary disease; COX: cytochrome c oxidase; COXact/Prot: COX activity per proteins; CS: citrate synthase; ECT: electron transport chain; INR: international normalized ratio; mOD: milli-optical density; mtDNA: mitochondrial deoxyribonucleic acid; OR: odds ratio; OXPHOS: oxidative phosphorylation; PaO_2_/FIO_2_: pressure of arterial oxygen/fraction inspired oxygen; rRNA: ribosomal RNA; SOFA: sepsis-related organ failure assessment score; tRNA: transfer RNA.

## Competing interests

The authors declare that they have no competing interests.

## Authors’ contributions

LLo and ERP have designed the study and drafting the manuscript. LLo, MMM, ELG, RI, JSV, JB, LLa, CD, JM and ERP have participated in the acquisition of data. LLo, AJ and ERP have performed the analysis and interpretation of data. All authors have revised the manuscript critically for important intellectual content and approved the final version of manuscript for publication.
